# Antiarrhythmic Effects of SGLT2 Inhibitors on Supraventricular Tachyarrhythmias in Patients with HFrEF

**DOI:** 10.3390/jcm14030786

**Published:** 2025-01-25

**Authors:** Lyuboslav Katov, Jonas Rostan, Yannick Teumer, Federica Diofano, Carlo Bothner, Wolfgang Rottbauer, Karolina Weinmann-Emhardt

**Affiliations:** Ulm University Heart Center, Ulm University, Albert-Einstein-Allee 23, 89081 Ulm, Germany

**Keywords:** SGLT2 inhibitor, ventricular tachycardia, supraventricular tachycardia, HFrEF, ICD, CRT-D

## Abstract

**Background**: In recent years, sodium-glucose cotransporter-2 (SGLT2) inhibitors have demonstrated significant cardiovascular and renal benefits in patients with heart failure (HF), in addition to their established antidiabetic effects. However, their role in arrhythmia prevention remains unclear. This study aimed to assess the effect of SGLT2 inhibitors on the incidence of supraventricular tachycardia (SVT) and ventricular tachycardia (VT) in patients with HF with reduced ejection fraction (HFrEF) during an extended follow-up period. **Methods**: This retrospective cohort study was conducted between January 2019 and November 2024 at the Ulm University Heart Center. All patients exhibited severely reduced left ventricular function and underwent primary prophylactic implantable cardioverter-defibrillator (ICD) implantation. Half of the cohort initiated SGLT2 inhibitor therapy alongside optimal medical HF treatment (the SGLT2 group). Patients were followed for approximately three years (846.2 ± 520.0 days) and the incidence of SVT and VT was analyzed using intracardiac Holter records of the ICD. **Results**: The study population consisted of 78 patients with a mean age of 66.6 ± 12.9 years. Over the follow-up period, a significant prolongation in the time to first occurrence of SVT was observed in the SGLT2 group (Log-Rank *p* = 0.03), suggesting a potential protective effect of SGLT2 inhibitors. However, regarding VT, additional SGLT2 inhibitor therapy did not show an additional benefit to optimal medical HF treatment. **Conclusions**: This study suggests that SGLT2 inhibitors may play a beneficial role in reducing the incidence of SVT in patients with HFrEF. These results highlight the importance of further investigating the antiarrhythmic potential of SGLT2 inhibitors through large-scale, prospective studies to better understand their clinical implications and mechanisms of action.

## 1. Introduction

Approximately 64 million people globally are estimated to suffer from heart failure (HF) [1,2], with a prevalence reaching up to 4% in Western countries [3]. The 1-year mortality rate for chronic HF ranges between 15% and 30% [2,4]. Arrhythmias represent a frequent and significant complication in HF, often exacerbating its progression or serving as a contributing factor to its development. These arrhythmias may originate from either the atrial or ventricular myocardium, presenting asymptomatically or with a broad spectrum of clinical symptoms [5].

Current guidelines recommend the early initiation of renin–angiotensin–aldosterone system (RAAS) inhibitors, beta-blockers, and mineralocorticoid receptor antagonists (MRAs) in the management of heart failure with reduced ejection fraction (HFrEF) [6,7]. Cardiac resynchronization therapy (CRT) has been shown to improve cardiac function and alleviate symptoms by restoring ventricular synchrony through the use of a three-lead device [6,8]. Landmark trials, such as MADIT and SCD-HeFT, have demonstrated survival benefits associated with implantable cardioverter-defibrillators (ICDs) in ischemic HF, though results in non-ischemic HF remain mixed [6,9,10,11,12]. Sodium-glucose cotransporter-2 (SGLT2) inhibitors were first recommended for HFrEF in the 2021 ESC guidelines [6]. Initially developed for glycemic control in diabetes, SGLT2 inhibitors have since been recognized for their cardiovascular and renoprotective properties. Trials in patients with type 2 diabetes have consistently demonstrated improved cardiovascular outcomes with these agents [13,14,15,16], prompting further exploration into their potential benefits for non-diabetic HF patients. While the antidiabetic mechanisms of SGLT2 inhibitors are well established, their precise mechanisms of cardioprotection and nephroprotection remain an area of active investigation. These drugs function by increasing renal glucose excretion, thereby lowering blood glucose levels and exerting various systemic effects. Lopaschuk and Verma have proposed 18 distinct mechanisms underlying their benefits, suggesting a multifactorial mode of action [17]. The DAPA-HF trial demonstrated that dapagliflozin significantly reduced both hospitalizations and cardiovascular mortality. A subgroup analysis in the patients with ICD/CRT-D revealed a reduction in life-threatening ventricular tachycardia (VT) and sudden cardiac death (SCD) [18,19,20]. Similarly, empagliflozin, approved for HFrEF, demonstrated in the EMPEROR-Reduced trial a significant reduction in hospitalizations and deaths compared to the placebo, with shorter times to first hospitalization and fewer total hospitalizations [21]. A meta-analysis of the DAPA-HF and EMPEROR-Reduced trials revealed a 13% reduction in all-cause mortality and a 25% reduction in cardiovascular death and hospitalization with SGLT2 inhibitors [22]. Other SGLT2 inhibitors, such as Canagliflozin, has been shown to significantly reduce cardiovascular death, nonfatal myocardial infarction, and stroke in type 2 diabetes patients, while also alleviating symptoms of HF [13,23]. A Phase 3 trial is currently evaluating the effects of ertugliflozin on ventricular arrhythmias in ICD/CRT-D patients [24].

SGLT2 inhibitors have emerged as a cornerstone in the treatment of HF, offering significant reductions in hospitalizations and mortality. This study aims to evaluate whether SGLT2 inhibitors can modulate the occurrence of VT or supraventricular tachycardias (SVTs) in HFrEF patients with an implanted defibrillator.

## 2. Materials and Methods

### 2.1. Patient Selection

This study analyzed patients who underwent ICD or CRT-D implantation at the University Heart Center Ulm between January 2019 and May 2022, with follow-up data collected until November 2024. Patients selected for analysis received devices for primary prevention according to guidelines, excluding those with prior VT (secondary indication) or with only mild left ejection function impairment. Exclusions were applied to ensure a homogenous study population and to minimize potential confounding factors that could bias the results. Patients without follow-up data were excluded because the lack of longitudinal data would hinder the ability to assess the occurrence and timing of arrhythmic events, which was a primary focus of this study. Patients with subcutaneous ICDs were excluded because these devices lack the capability to provide continuous intracardiac Holter monitoring, which is critical for the detailed detection and analysis of both supraventricular and ventricular arrhythmias. Additionally, patients who underwent major cardiac interventions within 90 days prior to the observation period were excluded to avoid confounding effects related to acute recovery or procedural outcomes. These interventions included percutaneous coronary intervention (PCI), coronary artery bypass grafting (CABG), percutaneous catheter-based heart valve repair, ablation for cardiac arrhythmias, and surgical valve procedures, all of which are known to significantly alter cardiac function, arrhythmia risk, and heart failure progression in the short term [25,26]. By excluding these patients, we aimed to isolate the effects of SGLT2 inhibitors on arrhythmic outcomes and to maintain consistency in the baseline clinical status of the study cohort. Data were extracted from medical records at the University Hospital Ulm, including discharge summaries, catheterization reports, device interrogations, and electrocardiograms (ECGs). Ethical approval was obtained from the local Ethics Committee of Ulm University, in accordance with the principles outlined in the Declaration of Helsinki (protocol 324/16, 12 October 2016).

### 2.2. Medical Heart Failure Treatment

All patients were on optimal medical HF treatment for at least 90 days prior to study inclusion. Each patient received a renin–angiotensin–aldosterone system (RAAS) blockade, which included angiotensin-converting enzyme inhibitors (ACE inhibitors), angiotensin II type 1 receptor blockers (AT1 receptor blockers), or angiotensin receptor–neprilysin inhibitors (ARNIs), in addition to MRAs and beta-blockers at the maximum individually tolerated clinical dosage. Half of the cohort was treated with SGLT2 inhibitors.

### 2.3. Follow-Up Visit

For patients receiving SGLT2 inhibitors, follow-up commenced at the time of drug initiation and ended upon discontinuation. For patients not on SGLT2 inhibitors, the observation period spanned from the time of device implantation to the last follow-up. All patients were monitored from the point of enrollment, with follow-up visits scheduled every 6 months in the outpatient clinic. Each follow-up visit included a detailed assessment of medical history, symptoms, a 12-lead ECG, transthoracic echocardiography, device interrogation with analysis of arrhythmic events from the implanted Holter ECG, and a review of current medications. Device-recorded arrhythmias identified during follow-up were categorized as sustained or non-sustained VT and SVT. Sustained VT was defined as episodes lasting more than 30 s or requiring intervention to terminate, while non-sustained VT was defined as episodes lasting less than 30 s and terminating spontaneously. SVT was defined as sustained episodes lasting longer than 30 s with atrial activity exceeding ventricular activity on intracardiac ECG.

### 2.4. Statistics

Data were processed in SPSS (version 29.0). Categorical variables were analyzed using Chi-square or Fisher’s exact tests, and continuous variables with t-tests or Mann–Whitney U tests as appropriate. Survival analyses employed Kaplan–Meier curves with group comparisons via Log Rank tests. Binary logistic regression was performed, and all variables with a *p*-value < 0.30 in the univariate analysis were included in the multivariate analysis. Odds ratios (OR) with corresponding 95% confidence intervals (CIs) were calculated for each variable to assess their potential as predictors. SGLT2 inhibitors were used in the multivariate model regardless of its statistical significance in the univariate analysis. With an alpha error of 0.05 and a desired power of 0.8, the estimated sample size required to detect significant differences was 190 patients for VT (95 per group) and 324 patients for SVT (162 per group). A *p*-value of <0.05 was considered statistically significant.

## 3. Results

### 3.1. Baseline Characteristics

A total of 78 patients were evaluated, with 39 patients receiving SGLT2 inhibitors and another 39 patients not undergoing SGLT2 inhibitor therapy. The mean age was 66.6 ± 12.9 years, and the majority of the patients were male (85.9%). Coronary artery disease was previously diagnosed in 62.0 (79.5%) patients. Type 2 diabetes mellitus was more frequently observed in the SGLT2 inhibitor group (*p* = 0.01), while a history of nicotine abuse was more common among patients without SGLT2 inhibitor therapy (*p* = 0.01). At the beginning of enrollment, atrial fibrillation (AF) was previously diagnosed in 27.0 patients (34.6%) with an atrial tachycardia/atrial fibrillation (AT/AF) burden recorded at 2.9 ± 4.3 min per day. Prior pulmonary vein isolation was more frequently observed in the SGLT2 inhibitor group (*p* = 0.02). No significant differences were noted in other comorbidities (Table 1).

Chronic kidney disease was previously diagnosed in 66.0 (84.6%) patients, with potassium levels at the time of administration of 4.4 ± 0.5 mmol/L. The mean NT-proBNP level was 2670.3 ± 6152.8 pg/mL.

### 3.2. Medication at the Beginning of Enrollment

At the start of enrollment, 56.0 (71.8%) of the patients were on optimal medical HF treatment, including RAAS blockade with ACE inhibitors, AT1 receptor blockers, or ARNIs, in addition to MRAs and beta-blockers. Loop diuretics were administered to 46 (59.0%) patients, with no statistically significant differences between the two groups (*p* = 0.17). Two patients (2.6%) were treated with amiodarone. No statistically significant differences were detected between the groups (Table 2).

### 3.3. Follow-Up

The follow-up period was on average 846.2 ± 520.0 days. Except for the therapy with SGLT2 inhibitors, no statistically significant differences were observed between the two groups in the medications at the end of the follow-up period (Table 3).

At the end of the follow-up period, the groups showed no differences in the number of arrhythmic episodes observed (Table 4).

#### 3.3.1. Follow-Up on VT Events

**Figure 1:**
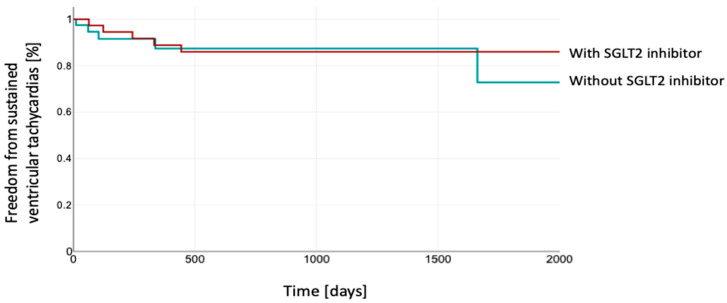
Kaplan–Meier estimation of freedom from sustained VTs between both groups. The patients depicted in red were treated with SGLT2 inhibitor, while those shown in green were without SGLT2 inhibitor. SGLT2, sodium–glucose co-transporter 2.

The follow-up analysis revealed no significant differences in the time to occurrence of sustained VT episodes between patients with or without SGLT2 inhibitor treatment (Log Rank 0.99) (Figure 1).

**Figure 2:**
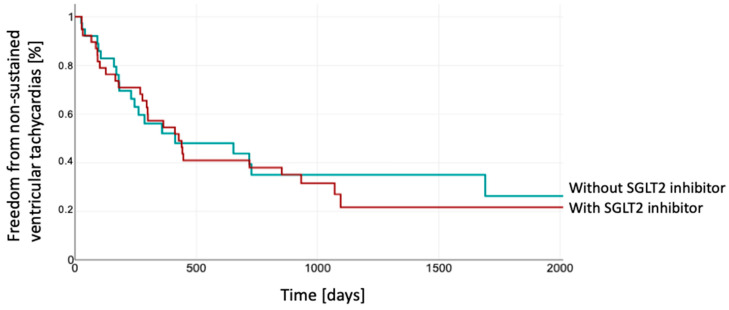
Kaplan–Meier estimation of freedom from non-sustained VTs between both groups. The patients depicted in red were treated with SGLT2 inhibitor, while those shown in green were without SGLT2 inhibitor. SGLT2, sodium–glucose co-transporter 2.

Furthermore, there were no statistically relevant differences in the time to occurrence of episodes with non-sustained VT (Log Rank 0.74) (Figure 2).

#### 3.3.2. Follow-Up on SVT Events

**Figure 3:**
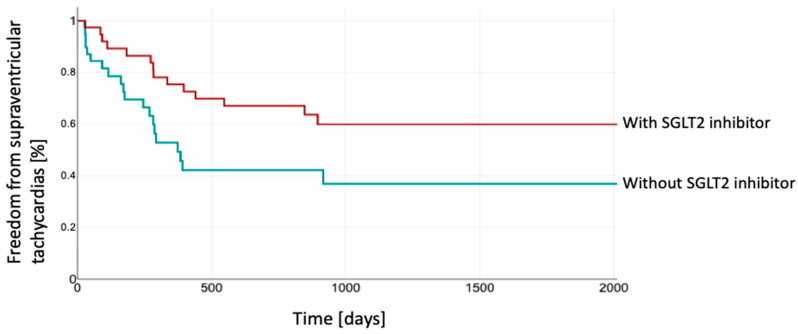
Kaplan–Meier estimation of freedom from SVTs between both groups. The patients depicted in red were treated with SGLT2 inhibitor, while those shown in green were without SGLT2 inhibitor. SGLT2, sodium–glucose co-transporter 2.

In the group receiving SGLT2 inhibitor therapy, a longer period of freedom from SVTs was observed (Log Rank 0.03) (Figure 3).

### 3.4. Predictor Models for VT Events

Binary logistic regression with selected baseline parameters identified no statistically significant predictors in the group of patients with sustained VT. Coronary artery disease was a statistically significant predictor in the group of patients with non-sustained VT (OR 3.25 (95% CI 1.04–10.18); *p* = 0.04). The use of SGLT2 inhibitors, along with other parameters, showed no significant differences (Table 5).

In the multivariate analysis, including all parameters with a *p*-value < 0.30 from the binary logistic regression, arterial hypertension showed a borderline significance (OR 0.24 (95% CI 0.06–0.99); *p* = 0.05), while coronary artery disease was identified as a significant predictor for non-sustained VT (OR 4.99 (95% CI 1.11–22.46); *p* = 0.04). No significant predictors were found in patients with sustained VT (Table 6).

### 3.5. Predictor Models for SVT Events

Binary logistic regression with selected baseline parameters revealed no relevant factors as potential predictors for the occurrence of SVT. Patients’ age demonstrated a statistical trend but did not reach the level of significance (OR 0.97 (95% CI 0.93–1.00); *p* = 0.08). The admission of SGLT2 inhibitors, as well as the other parameters, showed no statistically significant differences (Table 7).

In the multivariate analysis, including all parameters with a *p*-value < 0.30 from the binary logistic regression, no statistically significant predictors were identified (Table 8).

## 4. Discussion

### 4.1. Antiarrhythmic Mechanisms of SGLT2 Inhibitors and Aim of This Study

In addition to their antidiabetic and nephroprotective effects, SGLT2 inhibitors have gained significant attention for their cardiovascular benefits. They may also influence arrhythmias through structural and functional cardiac modifications. Preclinical and clinical studies suggest that SGLT2 inhibitors improve cardiac remodeling, reduce fibrosis, and enhance ventricular function [27,28,29]. Studies in animal models and humans indicate a potential decrease in sympathetic activity with SGLT2 inhibitor use, which could reduce arrhythmia risk [25,30,31]. Additionally, the stabilization of ion channel activity and calcium homeostasis has been investigated, helping to mitigate the proarrhythmic changes at the cellular level [32,33].

VTs, SVTs, and SCD are significant risks in patients with HFrEF, contributing substantially to mortality. Prophylactic defibrillators and medications like beta-blockers, MRAs, and RAAS blockers are recommended to mitigate this risk [6,26,34]. The reduction in hospitalizations and cardiovascular mortality in patients with HFrEF, compared to the placebo, was demonstrated in the EMPEROR-Reduced and DAPA-HF trials [18,19,20,21,22]. A recent meta-analysis further revealed that dapagliflozin significantly reduces the risk of AF, particularly in patients with diabetes [35]. This study aimed to evaluate the potential impact of SGLT2 inhibitors on the occurrence of VTs and SVTs in patients with HFrEF and an implanted defibrillator.

### 4.2. Impact of SGLT2 Inhibitors on VT

In recent years, extensive information has emerged regarding the impact of SGLT2 inhibitors on VTs. Meta-analyses have yielded varying results regarding their impact on ventricular arrhythmias. Li et al. reported a modest reduction in the risk of VT with SGLT2 inhibitors in a broad patient population, including individuals with HF, diabetes, or chronic kidney disease [36]. Other studies show significant reductions in the risk of SCD, but the effects on VT incidence remain inconsistent [37]. Some analyses suggest potential antiarrhythmic benefits, particularly in high-risk patients [19,38,39]. Though these findings require further validation through targeted research. Ongoing large-scale trials, such as EMPA-ICD and ERASe, aim to provide further insight into the antiarrhythmic effects of SGLT2 inhibitors [24,40].

In our study cohort, approximately one-tenth of the patients experienced sustained VTs during the follow-up period, while just over half had non-sustained VTs. The findings showed no significant improvement in VT-free survival among patients on SGLT2 inhibitors, both in the sustained and non-sustained VT groups.

### 4.3. Impact of SGLT2 Inhibitors on SVT

This study also assessed SVTs, revealing significantly lower SVT incidence among SGLT2 inhibitor users. However, previous studies, such as those by Chen et al. and Butt et al., found no significant differences in SVT or AF occurrence between treatment and control groups [41,42]. Nonetheless, some authors hypothesize that SGLT2 inhibitors may indirectly influence arrhythmogenesis by reducing mortality and composite arrhythmia endpoints [42,43]. Meta-analyses similarly yield conflicting results. Fernandes et al. and Wang et al. reported significant reductions in atrial arrhythmias with SGLT2 inhibitors, particularly dapagliflozin, although this was not consistently observed across all drugs [37,44]. Subgroup analyses restricted to HF patients often fail to demonstrate significant effects, suggesting further research is needed to confirm clinical relevance.

### 4.4. Multivariate Analyses

For sustained VT, no statistically significant predictors were found in either binary logistic regression or multivariate analysis. In patients with non-sustained VT, coronary artery disease emerged as a significant predictor in both analyses, which may reflect the influence of ischemic versus non-ischemic cardiomyopathy. This represents a potential confounder for VT and should be acknowledged as a limitation of this study. For SVT, neither analysis identified statistically significant predictors.

The use of SGLT2 inhibitors showed no relevant associations with VT or SVT events. These findings highlight the complexity and multifactorial nature of arrhythmogenesis in both VT and SVT, emphasizing the necessity for further research to uncover the underlying mechanisms and identify more reliable and clinically relevant risk factors for these arrhythmias.

### 4.5. Limitations

This study’s retrospective nature limits its ability to establish causality. The small sample size reduces statistical power, potentially obscuring true effects. The power analysis indicates that the study was underpowered to detect significant differences in VT and SVT. The findings for VT are limited in their interpretability, and the discussion refrains from making definitive conclusions regarding these outcomes, as the benefit remains unclear, highlighting the need for a larger sample size to achieve sufficient power. Despite the study’s limited power, a significant difference was observed for SVT, which strengthens the validity of this finding within the study’s framework. Given the highly selective cohort and the extensive screening of patients, larger multicenter studies with adequate power are required to confirm these results. Ongoing studies like EMPA-ICD and ERASe are expected to provide crucial insights, improving understanding of how SGLT2 inhibitors influence arrhythmogenesis and SCD risk in HF patients [24,40].

## 5. Conclusions

In this study, SGLT2 inhibitor therapy was associated with a significant reduction in the incidence of SVT, as demonstrated by continuous monitoring using implantable holter recordings over a 3-year follow-up period. The effect on VT remains inconclusive. These findings emphasize the need for further large-scale, prospective trials to better understand these effects and their underlying mechanisms.

## Figures and Tables

**Table 1:** Baseline characteristics of the patients within both groups.

Baseline Characteristics	All Patients (*n* = 78)	Patients with SGLT2 Inhibitor (*n* = 39)	Patients Without SGLT2 Inhibitor (*n* = 39)	*p*-Value
Age [years], mean ± SD	66.6 ± 12.9	65.9 ± 12.3	67.3 ± 13.7	0.63
Male, *n* (%)	67.0 (85.9)	34.0 (87.2)	33.0 (84.6)	0.74
BMI [kg/m^2^], mean ± SD	28.3 ± 5.6	28.5 ± 6.6	28.3 ± 4.6	0.81
Arterial hypertension, *n* (%)	54.0 (69.2)	26.0 (66.7)	28.0 (71.8)	0.62
Type II diabetes mellitus, *n* (%)	20.0 (25.6)	15.0 (38.5)	5.0 (12.8)	0.01
Nicotine abuse, *n* (%)	39.0 (50.0)	14.0 (35.6)	25.0 (64.1)	0.01
Hyperlipoproteinemia, *n* (%)	59.0 (75.6)	29.0 (74.4)	30.0 (76.9)	0.79
Coronary artery disease, *n*(%)	62.0 (79.5)	33.0 (84.6)	29.0 (74.4)	0.26
Atrial fibrillation, *n* (%)	27.0 (34.6)	11.0 (28.2)	16.0 (41.0)	0.23
Prior PVI, *n* (%)	8.0 (10.3)	7.0 (17.9)	1.0 (2.6)	0.02
Prior AV node ablation, *n* (%)	3.0 (3.9)	2.0 (5.1)	1.0 (2.6)	0.57
Prior CTI ablation, *n* (%)	2.0 (2.6)	2.0 (5.1)	0	0.15

AV, atrioventricular; BMI, Body Mass Index; CTI, cavotricuspid isthmus; PVI, pulmonary vein isolation; SD, standard deviation; SGLT2, sodium-glucose co-transporter 2.

**Table 2:** Medication of the patients at the beginning of enrollment within both groups.

Medication at the Beginning of Enrollment	All Patients (*n* = 78)	Patients with SGLT2 Inhibitor (*n* = 39)	Patients Without SGLT2 Inhibitor (*n* = 39)	*p*-Value
Beta-blocker, *n* (%)	75.0 (96.2)	38.0 (97.4)	37.0 (94.9)	0.56
RAAS-blocker, *n* (%)	77.0 (98.7)	39.0 (100)	38.0 (97.4)	0.31
MRA, *n* (%)	59.0 (75.6)	31.0 (79.5)	28.0 (71.8)	0.42
Loop diuretics, *n* (%)	46.0 (59.0)	20.0 (51.3)	26.0 (66.7)	0.17
Amiodarone, *n* (%)	2.0 (2.6)	1.0 (2.6)	1.0 (2.6)	0.56

MRA, mineralocorticoid receptor antagonist; RAAS, renin–angiotensin–aldosterone system.

**Table 3:** Medication of the patients at the end of the follow-up period within both groups.

Medication at the End of the Follow-Up Period	All Patients (*n* = 78)	Patients with SGLT2 Inhibitor (*n* = 39)	Patients Without SGLT2 Inhibitor (*n* = 39)	*p*-Value
Beta-blocker, *n* (%)	77.0 (98.7)	39.0 (100)	38.0 (97.4)	0.56
RAAS-blocker, *n* (%)	75.0 (96.2)	36.0 (92.3)	39.0 (100)	0.15
MRA, *n* (%)	60.0 (76.9)	31.0 (79.5)	29.0 (74.4)	0.59
Loop diuretics, *n* (%)	43.0 (55.1)	18.0 (46.2)	25.0 (64.1)	0.11
Amiodarone, *n* (%)	3 (4.0)	2.0 (5.1)	1.0 (2.6)	0.56

MRA, mineralocorticoid receptor antagonist; RAAS, renin–angiotensin–aldosterone system.

**Table 4:** Events at the end of the follow-up period within both groups.

Event	All Patients (*n* = 78)	Patients with SGLT2 Inhibitor (*n* = 39)	Patients Without SGLT2 Inhibitor (*n* = 39)	*p*-Value
Sustained ventricular tachycardias, *n* (%)	10.0 (12.8)	5.0 (12.8)	5.0 (12.8)	1.00
Non-sustained ventricular tachycardias, *n* (%)	47.0 (60.3)	27.0 (69.2)	20.0 (51.3)	0.10
Supraventricular tachycardias, *n* (%)	34.0 (43.6)	14.0 (35.9)	20.0 (51.3)	0.17

**Table 5:** Binary logistic regression for the sustained and non-sustained VT patient groups.

	Coefficient B	SE	z-Value	*p*-Value	Odds Ratio	95% CI
Sustained VT						
Age	0.03	0.03	1.08	0.28	1.03	0.97–1.1
Arterial hypertension	0.65	0.83	0.78	0.44	1.91	0.37–9.77
Nicotine abuse	0.46	0.69	0.67	0.50	1.59	0.41–6.15
Hyperlipoproteinemia	19.99	6764.51	0	0.99	Not applicable *	Not applicable *
Type II diabetes mellitus	0.25	0.74	0.34	0.74	1.29	0.30–5.53
FH for CV diseases	0.25	0.74	0.34	0.74	1.29	0.30–5.53
Atrial fibrillation	1.21	0.70	1.74	0.08	3.36	0.86–13.16
No prior PVI	−0.95	0.90	1.06	0.29	0.39	0.07–2.25
Coronary artery disease	19.93	7371.46	0	0.99	Not applicable *	Not applicable *
CABG	0.23	0.22	1.05	0.29	1.26	0.82–1.95
SGLT2 inhibitors	0	0.68	0	1.00	1	0.27–3.77
Non-sustained VT						
Age	0.01	0.02	0.56	0.58	1.01	0.98–1.05
Arterial hypertension	−0.66	0.53	1.26	0.21	0.51	0.18–1.44
Nicotine abuse	−0.32	0.46	0.69	0.49	0.72	0.29–1.80
Hyperlipoproteinemia	−0.16	0.54	0.30	0.77	0.85	0.29–2.47
Type II diabetes mellitus	0.89	0.58	1.54	0.12	2.44	0.78–7,60
FH for CV diseases	0.89	0.58	1.54	0.12	2.44	0.78–7.60
Atrial fibrillation	−0.77	0.49	1.58	0.11	0.46	0.18–1.20
No prior PVI	−0.75	0.85	0.88	0.38	0.47	0.09–2.50
Coronary artery disease	1.18	0.58	2.03	0.04	3.25	1.04–10.18
CABG	0.10	0.20	0.51	0.61	1.11	0.75–1.65
SGLT2 inhibitors	0.76	0.47	1.61	0.11	2.14	0.85–5.39

* The high odds ratio indicates instability in the model, caused by perfect separation. CABG, coronary artery bypass grafting; CI, confidence interval; CV, cardiovascular; FH, family history; PVI, pulmonary vein isolation; SGLT2, sodium-glucose cotransporter-2; SE, standard error; VT, ventricular tachycardia.

**Table 6:** Multivariate analysis for the sustained and non-sustained VT patient groups.

	Coefficient B	SE	z-Value	*p*-Value	Odds Ratio	95% CI
Sustained VT						
Age	0.01	0.03	0.26	0.79	1.01	0.95–1.07
CABG	0.29	0.25	1.16	0.24	1.34	0.82–2.20
Atrial fibrillation	1.20	0.88	1.36	0.17	3.33	0.59–18.86
No prior PVI	−0.32	1.14	0.28	0.78	0.73	0.08–6.73
SGLT2 inhibitors	0.13	0.79	0.16	0.87	1.14	0.24–5.39
Non-sustained VT						
Arterial hypertension	−1.43	0.72	1.97	0.05	0.24	0.06–0.99
FH for CV diseases	0.65	0.64	1.02	0.31	1.92	0.55–6.74
Type II diabetes mellitus	0.82	0.66	1.24	0.21	2.27	0.62–8.31
Coronary artery disease	1.61	0.77	2.09	0.04	4.99	1.11–22.46
Atrial fibrillation	−0.34	0.54	0.63	0.53	0.71	0.25–2.05
SGLT2 inhibitors	0.34	0.53	0.63	0.53	1.40	0.49–3.97

CABG, coronary artery bypass grafting; CI, confidence interval; CV, cardiovascular; FH, family history; PVI, pulmonary vein isolation; SGLT2, sodium-glucose cotransporter-2; SE, standard error; VT, ventricular tachycardia.

**Table 7:** Binary logistic regression for the SVT patient group.

	Coefficient B	SE	z-Value	*p*-Value	Odds Ratio	95% CI
Age	−0.03	0.02	1.77	0.08	0.97	0.93–1.00
Arterial hypertension	−0.37	0.49	0.76	0.45	0.69	0.26–1.81
Nicotine abuse	−0.21	0.46	0.46	0.65	0.81	0.33–1.99
Hyperlipoproteinemia	0.08	0.53	0.15	0.88	1.08	0.38–3.08
Type II diabetes mellitus	0.78	0.55	1.40	0.16	0.46	0.15–1.36
FH for CV diseases	0.08	0.52	0.15	0.88	1.08	0.39–3.00
Atrial fibrillation	0.28	0.48	0.59	0.55	1.33	0.52–3.39
No prior PVI	0.29	0.75	0.39	0.70	1.33	0.31–5.77
Coronary artery disease	−0.64	0.57	1.14	0.26	0.53	0.17–1.60
CABG	−0.19	0.21	0.91	0.36	0.83	0.55–1.25
SGLT2 inhibitors	−0.63	0.46	1.36	0.17	0.53	0.21–1.32

CABG, coronary artery bypass grafting; CI, confidence interval; CV, cardiovascular; FH, family history; PVI, pulmonary vein isolation; SGLT2, sodium-glucose cotransporter-2; SE, standard error.

**Table 8:** Multivariate analysis for the SVT patient group.

	Coefficient B	SE	z-Value	*p*-Value	Odds Ratio	95% CI
Age	−0.03	0.02	1.57	0.12	0.97	0.93–1.01
Type II diabetes mellitus	−0.47	0.59	0.79	0.43	0.63	0.20–1.99
Coronary artery disease	−0.17	0.63	0.27	0.79	0.85	0.25–2.90
SGLT2 inhibitors	−0.58	0.5	1.14	0.25	0.56	0.21–1.51

CI, confidence interval; SGLT2, sodium-glucose cotransporter-2; SE, standard error.

## Data Availability

The data presented in this study are available on request from the authors. The data are not publicly available due to data privacy laws.
